# Targeted gender equality approach to improving childhood nutritional status in Cross River State, Nigeria: a community-based quasi-experimental study

**DOI:** 10.3389/fnut.2026.1873005

**Published:** 2026-08-20

**Authors:** Regina Idu Ejemot-Nwadiaro, Elizabeth Libuo-Beshel Nji, Peter Bassey Enyievi, Chinenye Blessing Onyekwelu, Maurice-Joel Ugbe Ugbe, Okechukwu Paul-Chima Ugwu

**Affiliations:** 1 Directorate of Research, Innovation, Consultancy and Extension, Kampala International University, Kampala, Uganda; 2 Department of Public Health, University of Calabar, Calabar, Nigeria; 3 Department of Public Health and Community Medicine, Kampala International University in Tanzania, Dar es Salaam, Tanzania; 4 Department of Publication and Extension, Kampala International University, Kampala, Uganda

**Keywords:** child nutrition, community-based intervention, dietary diversity, gender equality strategies, Nigeria, stunting

## Abstract

**Introduction:**

Childhood undernutrition remains a critical public health problem globally, with the highest prevalence concentrated in rural, resource-constrained settings across sub-Saharan Africa. Gendered asymmetries in food access, resource control, and household decision-making substantially exacerbate nutritional disparities. This study assessed the effectiveness of a targeted gender equality intervention, delivered over a 16-week (approximately 4-month) period, on dietary diversity and nutritional outcomes in children under 5 years in Cross River State, Nigeria.

**Methods:**

A community-based quasi-experimental study with a pre-test/post-test control group design was conducted, enrolling 200 child-caregiver pairs allocated equally to intervention and control groups drawn from two purposively selected, socio-demographically comparable Local Government Areas (LGAs). The intervention comprised nutrition education, women’s empowerment sessions, and structured male partner engagement. Baseline and end-line (16-week) data on child anthropometry and minimum dietary diversity (MDD) were analyzed using independent *t*-tests, Chi-square tests, and analysis of covariance (ANCOVA) adjusting for baseline scores, child age, and household size.

**Results:**

Stunting prevalence was approximately 36% in both groups at baseline. Over the 16-week study period, ANCOVA-adjusted marginal means at end-line revealed significantly greater improvements in the intervention group for height-for-age z-scores (HAZ: −0.89 vs. −1.51; F = 10.05, *p* = 0.002), weight-for-age z-scores (WAZ: 0.53 vs. 0.21; F = 12.6, *p* = 0.001), and MDD (4.19 vs. 3.06; F = 79.6, *p* < 0.001). Stunting prevalence fell from 36.5% to 23.5% in the intervention group over the study period, whilst rising from 34.2% to 50.6% in the control group. The proportion of children achieving adequate MDD increased from 41.2% to 78.8% in the intervention group across the 16 weeks, compared with 45.6% to 49.4% in the control group.

**Conclusion:**

A 16-week gender-transformative nutrition education and empowerment strategy significantly improved dietary diversity and anthropometric outcomes in children under 5 years. Integration of such approaches into national nutrition policy frameworks is warranted, with particular relevance to advancing Sustainable Development Goal targets on zero hunger, child health, and gender equality.

## Introduction

Undernutrition and food insecurity constitute enduring threats to the survival, productivity, and wellbeing of children globally, with suboptimal diets recognized as leading contributors to morbidity and mortality. Sub-Saharan Africa bears a disproportionate share of this burden (1–4). The consequences of undernutrition, including impaired cognitive development, reduced educational attainment, and diminished economic productivity, are most severe among children under 5 years of age (5–7). Globally, undernutrition accounts for nearly half of all deaths in this age group, with stunting (linear growth failure, defined as height-for-age z-score [HAZ] below minus two standard deviations of the World Health Organization [WHO] growth reference) and wasting (acute undernutrition, reflected in a low weight-for-height z-score [WHZ]) remaining persistent challenges in the region (4, 8). According to the Nigeria Demographic and Health Survey 2023–24, 40% of children under five in Nigeria are stunted, 8% are wasted, and 27% are underweight, reflecting entrenched food insecurity, gender disparities, and inadequate implementation of nutrition-sensitive interventions (4, 9). These indicators are considerably worse in rural and conflict-affected areas (4, 8).

Stunting was selected as the primary outcome measure for this study because it captures the cumulative, longer-term effect of chronic dietary and caregiving deficits, rather than the acute, more transient nutritional insults reflected in wasting, and is therefore a more biologically appropriate indicator of sustained gains attributable to a structured, caregiving-focused intervention (6, 8). Stunting is additionally the primary indicator against which the World Health Assembly Global Nutrition Targets and Nigeria’s National Multisectoral Plan of Action for Food and Nutrition are benchmarked, giving findings on this outcome direct relevance to national and global policy tracking (8, 9). Weight-for-age (WAZ, an index of underweight) and minimum dietary diversity (MDD, defined as consumption of foods from at least four of seven WHO/UNICEF-defined food groups in the preceding 24 h) were included as complementary outcomes because they are more sensitive to short-term dietary change and could therefore be expected to respond within the 16-week study window even where linear growth gains are only beginning to emerge.

Growing evidence indicates that improving child nutrition requires attention not only to food access but also to the socio-cultural determinants of malnutrition, particularly gender inequality in caregiving roles, household food allocation, resource control, and health-related decision-making (10–12). Doss et al. (13) demonstrated that the household share of food expenditure increases with women’s property ownership, whilst Seidu et al. (14) and Bagade et al. (15) have linked women’s decision-making autonomy to improved healthcare utilization and child health. Women with greater autonomy tend to consume more nutritious diets and to provide higher-quality food to their children (16); yet structural constraints on women’s time, autonomy, and resource access frequently limit their capacity to provide optimal nutrition (7, 10, 17). In settings where patriarchal norms prevail, discriminatory feeding practices and unequal caregiving burdens further compound child malnutrition (15, 18).

Despite this evidence base, few nutrition interventions have directly targeted the gendered determinants of undernutrition. A substantial body of literature underscores the value of gender-transformative approaches that empower women, engage men as active caregivers, and challenge harmful social norms to improve maternal and child health outcomes (2, 12, 19, 20). Schling and Pazos (21) estimated that a one percentage point increase in women’s empowerment in productive decisions is associated with approximately 0.93% growth in agricultural output. Combining nutrition education with male caregiver involvement, joint decision-making, and gender-responsive programming has been shown to improve dietary practices and child nutritional status across diverse low-resource settings (10, 20, 22).

This study addresses a critical evidence and implementation gap by evaluating the effect of a targeted gender equality and nutrition education intervention on dietary diversity and child nutritional outcomes in Cross River State, Nigeria, a context characterized by high child undernutrition, poverty, and entrenched gender inequality (1, 2, 9). Context-specific interventions of this nature are essential to achieving SDG 2 on zero hunger, SDG 3 on good health and wellbeing, and SDG 5 on gender equality (4). By embedding gender-responsive messaging within nutrition programming, this study contributes evidence for scalable strategies that address both the immediate and structural drivers of child malnutrition in resource-constrained settings.

**FIGURE 1:**
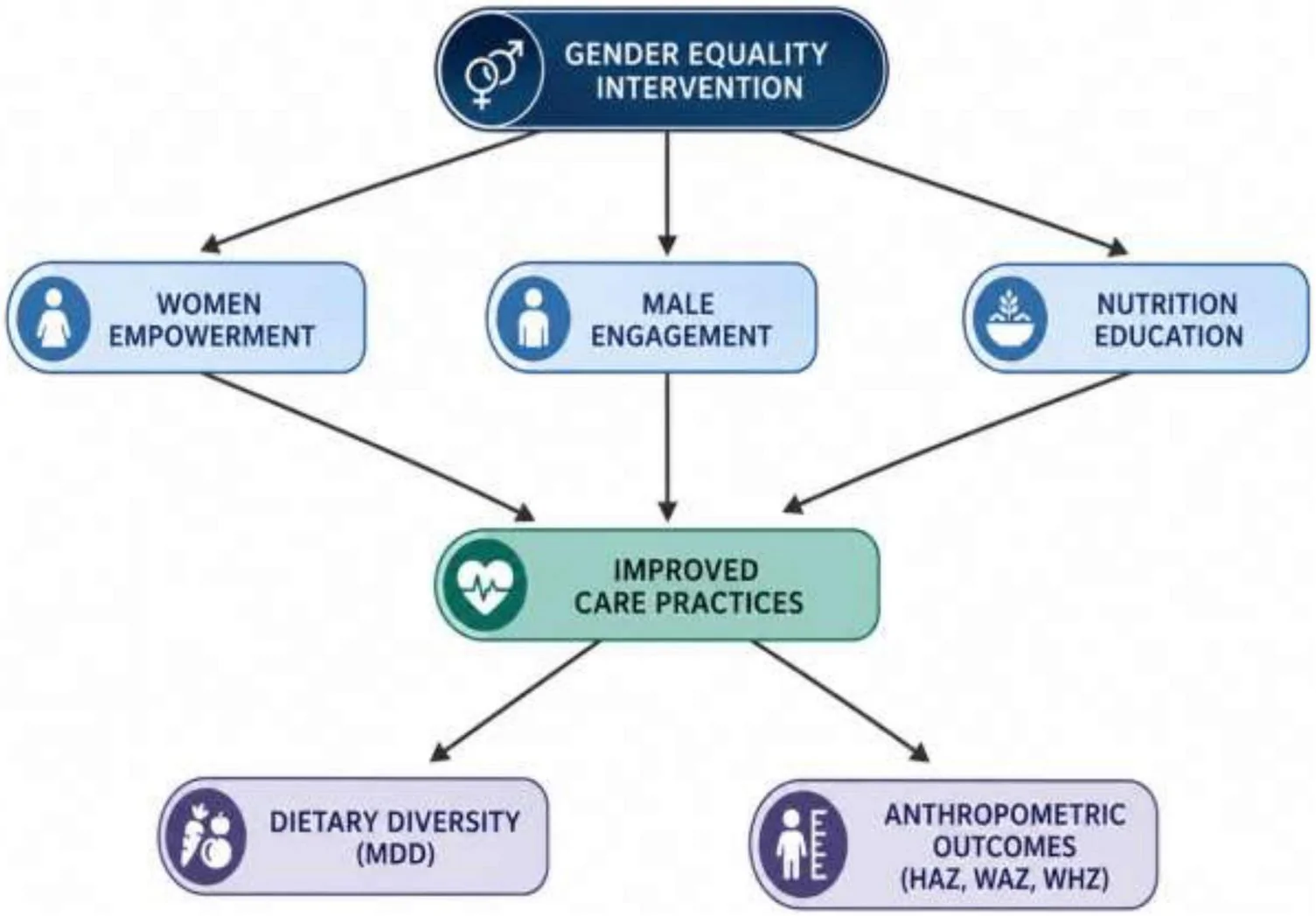
Conceptual framework. Pathways through which a gender-transformative intervention (women’s empowerment, male engagement, and nutrition education) influences child nutritional outcomes through improved caregiving practices, enhanced dietary diversity, and anthropometric status.

The study was conceptually grounded in an adapted pathway model (Figure 1), situated within UNICEF’s Conceptual Framework on Maternal and Child Nutrition (23) and informed by WHO Nutrition Guidelines (8). The model proposes that a gender-equality intervention acts through three interlinked channels – women’s empowerment, structured male partner engagement, and nutrition education – which jointly strengthen household care practices. These improved care practices are hypothesized to operate on child nutritional status through two proximal pathways: (i) enhanced dietary diversity, reflecting more varied and frequent access to nutrient-dense foods; and (ii) improved anthropometric status (HAZ, WAZ, WHZ), reflecting the cumulative biological effect of improved caregiving, feeding practice, and household resource allocation. This framework guided the selection of outcome measures and the choice of covariates in the statistical models described below.

## Methods

### Study design, setting, and participants

A community-based quasi-experimental study using a pre-test/post-test control group design was conducted in Cross River State (CRS), Nigeria. CRS comprises 18 Local Government Areas (LGAs) in the south-south geopolitical zone, with broadly comparable socio-demographic characteristics relevant to the study variables. Eligible participants were mothers or primary caregivers and their children under 5 years of age who had resided continuously in selected communities for at least 6 months prior to baseline data collection.

Children presenting with clinical signs of severe acute illness or congenital conditions affecting growth were excluded. Specifically, exclusion criteria comprised: congenital heart disease; cerebral palsy and other neuro-motor disabilities; Down syndrome and other chromosomal or genetic disorders known to affect growth; congenital hypothyroidism; cleft lip/palate with feeding difficulty; and acute conditions requiring hospital admission at the time of screening, including severe pneumonia, severe diarrhoeal disease with dehydration, and severe acute malnutrition with medical complications (8). Children meeting any of these criteria were referred to the nearest health facility for appropriate clinical management and were not enrolled.

### Justification for study site and group allocation

Two LGAs were purposively, rather than randomly, selected on the basis of four pre-specified criteria: (i) comparable baseline socio-demographic profile (population density, dominant livelihood, and reported stunting burden, drawn from LGA health records and the most recent Nigeria Demographic and Health Survey); (ii) logistic accessibility for repeated follow-up visits within the study timeframe; (iii) absence of a concurrent nutrition-specific or gender-equality programme that could confound the intervention effect; and (iv) documented willingness of local authorities and community structures to support programme delivery. One LGA was subsequently designated the intervention site and the other the control site by consensus of the research team, informed by the relative ease of programme roll-out (proximity to the implementing partner’s operational base) rather than by any characteristic of the eligible population; the two LGAs did not differ significantly on any measured baseline socio-demographic or nutritional indicator prior to allocation (Tables 1 and 2), which supports comparability at the point of assignment. We recognize, and discuss in the Limitations, that non-random allocation of LGAs (rather than of individuals) cannot fully exclude selection bias or unmeasured confounding at the community level; several individual-level baseline imbalances (caregiver age, household size, birth weight, and mid-upper arm circumference [MUAC]) did emerge despite LGA-level comparability, and these are addressed explicitly in the Statistical Analysis and Discussion sections below.

**TABLE 1:** Baseline socio-demographic characteristics of caregivers.

Characteristic	Intervention (*N* = 100) *n* (%)	Control (*N* = 100) *n* (%)	χ ^2^	*p*-value
Age of caregiver (years)			15.96	0.001*
<20	3 (3.0)	15 (15.0)		
20–29	51 (51.0)	49 (49.0)		
30–39	46 (46.0)	31 (31.0)		
≥40	0 (0.0)	5 (5.0)		
Marital status			1.39^1^	0.511
Married	75 (75.0)	68 (68.0)		
Single	21 (21.0)	28 (28.0)		
Separated/Divorced	4 (4.0)	4 (4.0)		
Educational level			1.77	0.123
Primary	5 (5.0)	8 (8.0)		
Secondary	57 (57.0)	57 (57.0)		
Tertiary	38 (38.0)	35 (35.0)		
Occupation			3.61	0.168
Unemployed	10 (10.0)	12 (12.0)		
Self-employed	76 (76.0)	82 (82.0)		
Formally employed	14 (14.0)	6 (6.0)		
Monthly household income (NGN)			0.47	0.804
Low (<30,000)	33 (33.0)	34 (35.8)		
Middle (30,001–90,000)	33 (33.0)	33 (34.7)		
High (>90,000)	34 (34.0)	28 (29.5)		
Household size			25.62	<0.001**
≤3 members	11 (11.0)	39 (39.0)		
4–6 members	74 (74.0)	58 (58.0)		
≥7 members	15 (15.0)	3 (3.0)		

^1^Fisher’s Exact statistic.

*p < 0.05;

**p < 0.001. NGN, Nigerian naira. Age and household-size categories have been consolidated from the originally recorded finer bands; χ^2^ statistics were recalculated for the consolidated categories shown.

**TABLE 2:** Baseline demographic and anthropometric characteristics of children.

Characteristic	Intervention (*N* = 100)	Control (*N* = 100)	Statistic	*p*-value
Age (months)			χ^2^ = 5.49	0.139
0 to <6	15 (15.0)	21 (21.0)		
6–11	7 (7.0)	13 (13.0)		
12–23	38 (38.0)	25 (25.0)		
24–59	40 (40.0)	41 (41.0)		
Sex			χ^2^ = 0.08	0.888
Male	50 (50.0)	48 (48.0)		
Female	50 (50.0)	52 (52.0)		
Birth weight			χ^2^ = 10.88	0.001*
<2.5 kg	27 (27.0)	8 (12.0)		
≥2.5 kg	73 (73.0)	88 (88.0)		
Height/length (cm), Mean ± SD	78.47 ± 17.41	75.65 ± 20.58	*t* = 1.05	0.297
Weight (kg), Mean ± SD	12.11 ± 3.39	11.31 ± 4.06	*t* = 1.51	0.133
MUAC (cm), Mean ± SD	14.81 ± 1.35	14.26 ± 1.32	*t* = 2.92	0.004*

MUAC, mid-upper arm circumference; SD, standard deviation.

*p < 0.05. The birth-weight imbalance (more low-birth-weight infants in the intervention arm) was not adjusted for in the primary ANCOVA models; see Statistical Analysis and Discussion.

Community entry and willingness to participate were established at two levels. At the administrative level, formal approval was obtained from LGA health authorities and traditional/community leadership structures prior to fieldwork, establishing programme legitimacy and access. At the individual level, household eligibility screening was followed by written (or witnessed thumbprint) informed consent from each caregiver, and participation remained voluntary throughout, with the right to withdraw at any stage without any effect on access to routine health services.

### Sample size determination

Using the formula for comparing two binomial proportions (24), a minimum of 200 caregiver-child pairs (100 per arm) was required to achieve 80% statistical power at the 5% significance level to detect a reduction in stunting prevalence from 32% in the control group (7) to 15% in the intervention group.

### Sampling and recruitment

Eligible households within each community were selected by simple random sampling. Given the participatory nature of the intervention, allocation concealment was not feasible; however, outcome assessment was standardized across both sites, with anthropometric and dietary diversity data collected by field teams following identical protocols in both arms. Community health workers assisted with participant recruitment and scheduled follow-up visits.

### Intervention

The intervention comprised three integrated components delivered over 16 weeks. The first was a targeted gender-equality nutrition education programme designed to improve maternal knowledge, dietary practices, and equitable household food allocation. The second was structured male partner engagement through participatory group sessions designed to challenge harmful gender norms and promote shared caregiving and food budgeting. The third was women’s leadership and peer support training to strengthen women’s capacity for household decision-making and resource allocation. The programme was delivered through structured group sessions, supervised home visits, and context-specific visual communication tools including flipcharts and locally adapted guides. The control group received no gender-equality-focused intervention during the study period.

The 16-week duration was informed by two considerations. First, prior gender-transformative and nutrition-education interventions of comparable intensity in low-resource settings have demonstrated measurable behavioral and dietary change within 12-to-20-week windows (20, 25), providing an evidence-based basis for the selected timeframe. Second, 16 weeks was judged sufficient to capture change in the more behaviourally responsive outcomes (dietary diversity and weight-based indices) while representing a realistic, fundable implementation period for a single programme cycle. We acknowledge, and discuss in the Limitations, that 16 weeks is a comparatively short window in which to detect the full biological effect of an intervention on linear growth (stunting), since HAZ responds more slowly than weight-based or dietary indices to improved caregiving and feeding practice; the magnitude of the HAZ effect observed here should accordingly be interpreted as an early signal, with longer follow-up required to confirm whether gains in linear growth are sustained or amplified over time.

### Data collection: anthropometric measurement

Anthropometric data were collected at baseline and at end-line, 16 weeks (approximately 4 months) after intervention commencement, by trained field workers using standardized WHO procedures (8). Body weight was measured to the nearest 0.1 kg using a calibrated digital weighing scale (SECA 874, seca gmbh, Hamburg, Germany); recumbent length (children under 24 months) or standing height (children 24 months and older) was measured to the nearest 0.1 cm using a portable infantometer/stadiometer (SECA 417, seca gmbh); and mid-upper arm circumference (MUAC) was measured to the nearest 0.1 cm using a UNICEF-standard, non-stretch, color-coded MUAC tape. Scales were calibrated daily against a known standard weight prior to fieldwork.

Measurement quality was assessed through a structured inter- and intra-observer reliability exercise. Prior to data collection, all anthropometrists underwent standardization training, after which each completed a set of repeated measurements on a common subsample of children, supervised by a senior anthropometrist. During fieldwork, a random 10% subsample of children was remeasured independently by a second anthropometrist (inter-observer reliability) and by the original anthropometrist on a separate occasion within 24 h (intra-observer reliability). Technical error of measurement (TEM) and the coefficient of reliability were calculated for height/length, weight, and MUAC following standard procedures (8); measurements were accepted where percentage TEM fell within WHO-recommended tolerance limits for each indicator, and repeated where this threshold was exceeded. This quality-control step directly supports the validity of the stunting, underweight, and wasting indicators derived from the anthropometric data.

### Data collection: infant and young child feeding (IYCF) and dietary diversity

Dietary diversity data were collected separately from anthropometric data, using a pre-tested, interviewer-administered semi-structured questionnaire adapted from the WHO Infant and Young Child Feeding (IYCF) indicator guide and the UNICEF Multiple Indicator Cluster Survey (MICS). Caregivers reported, by 24-h dietary recall, all foods and liquids consumed by the index child on the day and night preceding the interview. Minimum dietary diversity (MDD) was computed for children aged 6 to 59 months as consumption of foods from at least four of seven standard food groups within the preceding 24 h, consistent with the analytical approach of Raru et al. (22). Secondary outcomes included caregiver-reported changes in knowledge and attitudes regarding gender disparities in household food distribution.

### Statistical analysis

A dedicated statistical analysis plan was applied uniformly to both study arms. Data were entered into Microsoft Excel 2019, verified for completeness and range, and transferred to IBM SPSS Statistics Version 23 for analysis. Descriptive statistics (means, standard deviations, and proportions) summarized participant characteristics at baseline. Between-group comparisons of categorical variables were performed using the Chi-square test, with Fisher’s Exact Test applied where more than 20% of expected cell counts were below five; continuous variables were compared using independent-samples *t*-tests.

The primary analysis used analysis of covariance (ANCOVA) to estimate the adjusted effect of the intervention on end-line HAZ, WAZ, WHZ, and MDD. Each ANCOVA model included the corresponding baseline (pre-intervention) score of the same outcome variable as the principal covariate, together with child age and household size, which were retained *a priori* as covariates on the basis of their established association with child nutritional status in the literature (5, 26, 27). Estimated marginal means, F-statistics, partial eta squared (η^2^) as the effect-size estimate, and *p*-values are reported for each adjusted model; for comparison, the corresponding unadjusted between-group test (independent *t*-test or Chi-square, as appropriate) from the end-line descriptive tables is reported alongside each adjusted result, allowing readers to gauge the contribution of covariate adjustment to the observed effect (see Tables 5 and 6).

Several baseline characteristics caregiver age, household size, birth weight, and MUAC differed significantly between arms despite LGA-level comparability (Tables 1 and 2). Household size and child age were addressed directly through covariate adjustment in the ANCOVA models. Birth weight and caregiver age were not included as covariates in the primary models reported here, because doing so would require re-specification and re-estimation of the ANCOVA against the underlying dataset; this is flagged explicitly as a limitation, and we recommend that a sensitivity analysis incorporating birth weight and caregiver age as additional covariates be conducted and reported in any subsequent version or companion analysis, to confirm that the direction and significance of the intervention effect are robust to this adjustment. All inferences were evaluated at a 95% confidence interval with the significance threshold set at α = 0.05.

Anthropometric and dietary diversity analyses were restricted to children aged 6 to 59 months. Children with HAZ, WAZ, or WHZ below minus two standard deviations from the WHO growth reference were classified as stunted, underweight, or wasted, respectively. Z-scores were calculated using the WHO Anthro Calculator version 3.2.2.

### Ethical considerations

Ethical approval was obtained from the Health Research Ethics Committee of the University of Calabar Teaching Hospital, Calabar, Nigeria (Approval Number: UCTH/HREC/33/Vol.III/056). All procedures conformed to the ethical principles of the Declaration of Helsinki and Nigeria’s National Code of Health Research Ethics. Written informed consent was obtained from all participants prior to enrolment; where participants were not literate, the consent process was conducted in the local language and a thumbprint was obtained in lieu of a signature, with an impartial witness present. Participation was entirely voluntary, and the confidentiality and anonymity of all data were assured. No identifiable personal information was collected or published.

## Results

A total of 400 individuals were enrolled: 200 caregivers (100 per arm) and 200 children under 5 years (100 per arm). Two hundred children were assessed at baseline; of these, 85 in the intervention arm and 79 in the control arm were aged 6 to 59 months and were therefore eligible for the anthropometric and dietary diversity indicators reported below.

### Baseline socio-demographic characteristics of caregivers

Caregiver socio-demographic characteristics, presented using consolidated age and household-size categories for clarity, are summarized in Table 1. A statistically significant difference in age distribution was observed between the two groups (χ^2^ = 15.96, df = 3, *p* = 0.001), with a notably higher proportion of caregivers aged under 20 years in the control group (15.0% vs. 3.0%) and a higher proportion aged 40 years and above in the control group (5.0% vs. 0.0%). No significant differences were found in marital status (Fisher’s Exact χ^2^ = 1.39, *p* = 0.511), educational attainment (χ^2^ = 1.77, *p* = 0.123), or occupation (χ^2^ = 3.61, *p* = 0.168). The majority of caregivers in both groups had attained at least secondary-level education and were self-employed. Monthly household income was comparable across arms (*p* = 0.804). Household size, consolidated into three categories, differed significantly between groups (χ^2^ = 25.62, df = 2, *p* < 0.001), with smaller households (≤3 members) more prevalent in the control group (39.0%) than in the intervention group (11.0%), and larger households (≥7 members) more prevalent in the intervention group (15.0%) than in the control group (3.0%).

### Baseline demographic and anthropometric characteristics of children

Two hundred children were assessed at baseline, 100 per arm (Table 2). Age distribution did not differ significantly between groups (χ^2^ = 5.49, *p* = 0.139), with the largest proportion of children aged 24 to 59 months in both the intervention (40.0%) and control (41.0%) groups. Sex was similarly distributed across arms (χ^2^ = 0.08, *p* = 0.888). A significant difference in birth weight was observed (χ^2^ = 10.88, *p* = 0.001), with a higher proportion of low-birth-weight infants (<2.5 kg) in the intervention group (27.0%) than in the control group (12.0%); as noted in the Statistical Analysis section, this imbalance was not adjusted for in the primary ANCOVA models and is addressed as a limitation in the Discussion. Birth order did not differ significantly across arms (Fisher’s Exact χ^2^ = 6.83, *p* = 0.137). Mean height (78.47 ± 17.41 cm vs. 75.65 ± 20.58 cm; *t* = 1.05, *p* = 0.297) and weight (12.11 ± 3.39 kg vs. 11.31 ± 4.06 kg; *t* = 1.51, *p* = 0.133) were comparable. MUAC was significantly higher in the intervention group (14.81 ± 1.35 cm vs. 14.26 ± 1.32 cm; *t* = 2.92, *p* = 0.004).

### Nutritional status of children aged 6 to 59 months: baseline and end-line

Baseline and end-line nutritional status of children aged 6 to 59 months (intervention: *n* = 85; control: *n* = 79) are presented together in Table 3 to facilitate direct comparison of change over the 16-week study period. At baseline, mean HAZ (−1.27 ± 2.24 vs. −1.24 ± 2.06; *t* = −0.11, *p* = 0.913), WAZ (0.31 ± 1.70 vs. −0.18 ± 1.69; *t* = 1.84, *p* = 0.068), WHZ (1.13 ± 1.58 vs. 0.70 ± 1.62; *t* = 1.71, *p* = 0.089), stunting (36.5% vs. 34.2%; χ^2^ = 0.09, *p* = 0.870), underweight (11.8% vs. 13.9%; χ^2^ = 0.17, *p* = 0.679), and wasting (3.5% vs. 5.1%; χ^2^ = 0.24, *p* = 0.627) were statistically equivalent between arms, confirming baseline equivalence across all primary outcome measures. At the 16-week end-line, the intervention group showed significantly better mean HAZ (−0.90 ± 1.72 vs. −1.49 ± 1.76; *t* = 2.19, *p* = 0.027) and significantly higher mean WAZ (0.68 ± 0.99 vs. 0.04 ± 1.44; *t* = 3.31, *p* = 0.001) than the control group. Mean WHZ was higher in the intervention group (1.19 ± 1.49 vs. 0.73 ± 1.51) but the difference did not reach significance (*t* = 1.94, *p* = 0.055). Stunting prevalence fell to 23.5% in the intervention group by end-line, compared with a rise to 50.6% in the control group (χ^2^ = 12.9, *p* < 0.001). No child in the intervention group was classified as underweight at end-line, compared with 8.9% in the control group (Fisher’s Exact *p* = 0.005). Wasting prevalence remained low and statistically comparable across arms at end-line (2.1% vs. 1.9%; Fisher’s Exact *p* = 0.662).

**TABLE 3:** Nutritional status of children aged 6–59 months at baseline and 16-week end-line, by study arm.

Metric	Intervention baseline	Intervention end-line	Control baseline	Control end-line	End-line *p*-value
HAZ, Mean ± SD	−1.27 ± 2.24	−0.90 ± 1.72	−1.24 ± 2.06	−1.49 ± 1.76	0.027*
WAZ, Mean ± SD	0.31 ± 1.70	0.68 ± 0.99	−0.18 ± 1.69	0.04 ± 1.44	0.001*
WHZ, Mean ± SD	1.13 ± 1.58	1.19 ± 1.49	0.70 ± 1.62	0.73 ± 1.51	0.055
Stunted, *n* (%)	31 (36.5)	20 (23.5)	27 (34.2)	40 (50.6)	<0.001**
Underweight, *n* (%)	10 (11.8)	0 (0.0)	11 (13.9)	7 (8.9)	0.005*
Wasted, *n* (%)	3 (3.5)	2 (2.1)	4 (5.1)	2 (1.9)	0.662

HAZ, height-for-age z-score; WAZ, weight-for-age z-score; WHZ, weight-for-height z-score; SD, standard deviation. End-line *p*-values are unadjusted (independent *t*-test for continuous measures; Chi-square or Fisher’s Exact test for categorical measures) between intervention and control arms; corresponding ANCOVA-adjusted estimates are presented in Table 5.

*p < 0.05;

**p < 0.001.

### Minimum dietary diversity (MDD) among children aged 6 to 59 months: Baseline and end-line

Baseline and end-line dietary diversity are presented together in Table 4. At baseline, dietary diversity was similarly low across both groups, with no significant between-group differences (mean MDD score 2.67 ± 1.25 vs. 2.89 ± 1.16, *t* = −1.15, *p* = 0.254; adequate MDD 41.2% vs. 45.6%, χ^2^ = 0.32, *p* = 0.570). By the 16-week end-line, mean MDD score was significantly higher in the intervention group (4.15 ± 0.82 vs. 3.10 ± 1.07; *t* = 7.08, *p* < 0.001), and the proportion of children meeting the adequate MDD threshold was 78.8% in the intervention group compared with 49.4% in the control group (χ^2^ = 15.5, *p* < 0.001) an increase of 37.6 percentage points from baseline in the intervention group over the 16 weeks, versus 3.8 percentage points in the control group over the same period.

**TABLE 4:** Minimum dietary diversity (MDD) among children aged 6–59 months at baseline and 16-week end-line, by study arm.

IYCF indicator	Intervention baseline	Intervention end-line	Control baseline	Control end-line	End-line *p*-value
Mean MDD score	2.67 ± 1.25	4.15 ± 0.82	2.89 ± 1.16	3.10 ± 1.07	<0.001**
Adequate MDD (≥4 food groups), *n* (%)	35 (41.2)	67 (78.8)	36 (45.6)	39 (49.4)	<0.001**
Inadequate MDD (<4 food groups), *n* (%)	50 (58.8)	18 (21.2)	43 (54.4)	40 (50.6)	–

MDD, minimum dietary diversity; IYCF, infant and young child feeding; SD, standard deviation. End-line *p*-values are unadjusted between-arm comparisons; corresponding ANCOVA-adjusted estimates are presented in Table 6.

**p < 0.001.

### Intervention effect on anthropometric indices: adjusted and unadjusted comparison

Table 5 presents ANCOVA-adjusted end-line marginal means alongside the corresponding unadjusted between-group comparison from Table 3, so that the contribution of covariate adjustment (baseline score, child age, household size) can be assessed directly. For HAZ, the adjusted end-line marginal means were −0.89 in the intervention group and −1.51 in the control group (F = 10.05, *p* = 0.002; η^2^ = 0.059) a larger effect, in significance terms, than the corresponding unadjusted comparison (*p* = 0.027), consistent with adjustment for baseline HAZ and household composition sharpening the estimated intervention effect. For WAZ, adjusted marginal means were 0.53 and 0.21 in the intervention and control groups, respectively (F = 12.6, *p* = 0.001; η^2^ = 0.073), closely matching the unadjusted result (*p* = 0.001). The ANCOVA for WHZ revealed no statistically significant between-group difference (F = 2.03, *p* = 0.156), consistent with the non-significant unadjusted comparison (*p* = 0.055), indicating that the intervention did not produce a measurable effect on acute nutritional status within the 16-week study period after adjustment for baseline values.

**TABLE 5:** ANCOVA-adjusted and unadjusted intervention effects on anthropometric indices at end-line.

Index	Group	Baseline Mean ± SD	End-line Mean ± SD	Adjusted End-line Mean	F	η ^2^	Adjusted *p*	Unadjusted *p*
HAZ	Intervention	−1.27 ± 2.24	−0.90 ± 1.72	−0.89	10.05	0.059	0.002*	0.027*
	Control	−1.24 ± 2.06	−1.49 ± 1.76	−1.51				
WAZ	Intervention	0.31 ± 1.70	0.68 ± 0.99	0.53	12.6	0.073	0.001*	0.001*
	Control	−0.18 ± 1.69	0.04 ± 1.44	0.21				
WHZ	Intervention	1.13 ± 1.58	1.19 ± 1.49	0.99	2.03	N/A	0.156	0.055
	Control	0.70 ± 1.62	0.73 ± 1.51	0.94				

η^2^, partial eta squared (effect size). Adjusted models included baseline score of the same outcome, child age, and household size as covariates (see Statistical Analysis).

*p < 0.05. HAZ, height-for-age z-score; WAZ, weight-for-age z-score; WHZ, weight-for-height z-score; SD, standard deviation; N/A, not applicable.

### Intervention effect on minimum dietary diversity: adjusted and unadjusted comparison

ANCOVA results for MDD, adjusting for baseline dietary diversity score, child age, and household size, are presented in Table 6 alongside the corresponding unadjusted comparison. The adjusted end-line marginal mean was 4.19 in the intervention group compared with 3.06 in the control group (F = 79.6, *p* < 0.001; η^2^ = 0.331), closely consistent with the unadjusted comparison (*p* < 0.001). The large effect size indicates that the intervention produced a practically and statistically meaningful improvement in dietary diversity, substantially exceeding standard thresholds for clinical significance, and that this effect is robust to covariate adjustment.

**TABLE 6:** ANCOVA-adjusted and unadjusted intervention effects on minimum dietary diversity (MDD) in children aged 6–59 months.

Group	Baseline Mean ± SD	End-line Mean ± SD	Adjusted End-line Mean	F	η ^2^	Adjusted *p*	Unadjusted *p*
Intervention	2.67 ± 1.25	4.15 ± 0.82	4.19	79.6	0.331	<0.001**	<0.001**
Control	2.89 ± 1.16	3.10 ± 1.07	3.06		

η^2^, partial eta squared (effect size). Adjusted model included baseline MDD score, child age, and household size as covariates.

**p < 0.001. MDD, minimum dietary diversity; SD, standard deviation.

## Discussion

This study evaluated the effectiveness of a targeted gender equality intervention in improving nutritional status and dietary diversity among children under 5 years in Cross River State, Nigeria, over a 16-week implementation and follow-up period. The observed baseline stunting prevalence of approximately 36% in both arms aligns with the national estimate of 40% reported in the 2023–24 Nigeria Demographic and Health Survey (9), and considerably exceeds the WHO public health significance threshold of 20% (6, 8). These figures underscore the critical need for contextually tailored, structurally informed interventions to disrupt the intergenerational cycle of malnutrition in this setting.

The intervention produced statistically significant improvements in HAZ (adjusted *p* = 0.002) and WAZ (adjusted *p* = 0.001) after adjustment for baseline values, child age, and household size, with corresponding reductions in stunting and underweight prevalence over the 16-week period. These findings are consistent with evidence linking child nutritional outcomes to women’s dietary knowledge and empowerment (1, 19, 26, 28), equitable household resource allocation (13), and shared caregiving practices (10, 15, 20). They also corroborate prior evidence that gender-transformative and inclusive nutrition programmes yield significant anthropometric benefits for young children (10, 16, 29, 30).

### Biological and mechanistic pathways

The observed anthropometric and dietary gains are plausibly mediated through several interlinked pathways. First, at the household level, women’s empowerment and structured male engagement appear to have increased caregivers’ effective control over food purchasing and allocation decisions, translating into more frequent access to protein-rich and micronutrient-dense foods, as reflected in the marked rise in MDD (13, 19). Second, at the behavioral level, nutrition education combined with male partner involvement is associated with improved complementary feeding frequency and responsiveness, more consistent breastfeeding continuation, and reduced intra-household food-allocation inequity favoring adult males over young children, all of which act on growth via improved energy and micronutrient adequacy during the critical complementary feeding window (5, 25). Third, at the biological level, improved dietary diversity supplies the amino acid, iron, zinc, and vitamin A intake required to support insulin-like growth factor-1 (IGF-1) signaling and linear bone growth, while also reducing the frequency and severity of infections associated with micronutrient deficiency; this dual nutritional-immunological mechanism is consistent with the more rapid response observed in weight-based indices (WAZ, MDD) relative to the comparatively slower-responding linear growth indicator (HAZ) (5, 6). This distinction plausibly explains why WAZ and MDD showed larger, more consistent effects than HAZ within the relatively short 16-week window, and why the WHZ effect did not reach significance.

The baseline prevalence of inadequate MDD of approximately 59% reflects a substantial burden of micronutrient insufficiency in this population, although this figure is lower than the national average of 87% reported in the Nigeria Demographic and Health Survey 2023–24 (9). Following the intervention, the proportion of children achieving adequate MDD rose from 41.2% to 78.8% in the intervention group over the 16-week period, compared with a marginal change from 45.6% to 49.4% in the control group over the same period. These findings are consistent with evidence that dietary diversity is highly responsive to household-level, context-specific nutrition education and empowerment interventions (4, 22, 29, 31). Given that dietary diversity serves as a validated proxy measure for micronutrient adequacy (32), the magnitude of improvement observed carries clear implications for long-term child health and developmental outcomes.

These findings are congruent with comparable evidence from low- and middle-income countries. Aidam et al. (33) reported that a grandmother-inclusive IYCF programme in Sierra Leone achieved adequate MDD in 77.2% of infants aged 6 to 23 months in the intervention group compared with 51.8% in controls (*p* < 0.001). Sánchez-Encalada et al. (30) observed significant improvement in WAZ following a mother-focused educational intervention in Mexico, from −1.49 ± 0.65 at baseline to −1.19 ± 0.60 at end-line (*p* = 0.001). Edafioghor et al. (34) documented significant gains in maternal IYCF knowledge following nutrition education in Abakaliki, Nigeria, whilst Hien et al. (35) reported improved MDD and overall nutritional status in peri-urban Burkina Faso. Similar benefits of nutrition education for combating undernutrition have also been reported in a developing-country setting (36). The convergence of evidence across these diverse settings reinforces the proposition that addressing gendered barriers in caregiving and household resource allocation positively influences child feeding practices and growth trajectories (5, 25).

Taken together, these results support the UNICEF Conceptual Framework (23) and the study’s adapted pathway model (Figure 1), which identify gender equality as a critical enabling condition for addressing the underlying determinants of undernutrition. The integration of women’s empowerment, structured male caregiver engagement, and equitable food distribution within a unified programme package appears to have been instrumental in generating the observed improvements. There is growing evidence that embedding gender-transformative components into nutrition programming enhances programme effectiveness and scalability (20, 21, 35). The absence of a significant WHZ effect within the 16-week study period is itself informative, suggesting that acute nutritional status may respond more slowly, or require a longer or more intensive intervention, to yield measurable change; this observation merits attention as a design consideration in future research.

## Strengths, limitations, and interpretation caveats

This study is among the few quasi-experimental investigations in Nigeria and sub-Saharan Africa to rigorously evaluate the effects of a gender-transformative nutrition intervention on child dietary diversity and anthropometry using pre-specified outcome measures and ANCOVA adjustment for baseline covariates, which strengthens the internal validity of the estimated effects. Several limitations, however, warrant caution in interpretation. First, the quasi-experimental design, with purposive (non-random) allocation of LGAs rather than individuals, precludes full control for unmeasured confounding at both the community and individual level; as detailed in the Methods, the two LGAs were selected for comparability and were statistically equivalent on measured characteristics prior to allocation, but residual selection effects cannot be excluded. Second, despite this LGA-level comparability, individual-level baseline imbalances emerged in caregiver age, household size, birth weight, and MUAC; household size and child age were adjusted for in the ANCOVA models, but birth weight and caregiver age were not, and their potential influence on the observed intervention effects should be regarded as an open question pending sensitivity analysis. Third, the 16-week (4-month) end-line assessment is a comparatively short window for detecting the full biological effect of the intervention on linear growth, and limits conclusions regarding the long-term sustainability of the observed gains, particularly for HAZ and WHZ. Fourth, aggregate-level analysis may not fully capture within-group heterogeneity, and individual-level multivariate modeling using the full covariate set would strengthen future analyses of this dataset.

Standardized recruitment procedures, structured measurement quality-control (inter- and intra-observer reliability testing), and consistent outcome-assessment protocols across both sites helped to minimize systematic measurement bias, and the internal consistency, directionality, and magnitude of findings across multiple outcome measures support the credibility of the observed intervention effect. The interpretation is further strengthened by the alignment of these findings with prior evidence on the mediating roles of maternal agency, household size, and child age in nutritional outcomes (5, 26, 27). Future studies should incorporate extended follow-up beyond 16 weeks, individual-level multivariate modeling that includes birth weight and caregiver age as covariates, and cost-effectiveness analyses, to establish the scalability and sustained impact of this intervention model.

## Conclusion

This community-based quasi-experimental study provides compelling evidence that a targeted gender equality intervention, integrating women’s empowerment, gender-responsive nutrition education, and structured male caregiver engagement, significantly improved nutritional status and dietary diversity in children under 5 years in Cross River State, Nigeria, over a 16-week period. The intervention group experienced clinically meaningful reductions in stunting and underweight prevalence alongside substantial improvements in minimum dietary diversity. These outcomes affirm the importance of integrating gender-transformative approaches into child nutrition programming to enhance caregiving practices, improve dietary quality, and advance equitable health outcomes in low-resource settings. The findings are robust and consistent with global evidence for nutrition-sensitive interventions that address the structural determinants of malnutrition. Scaling such approaches across comparable settings represents a practical, evidence-informed, and policy-relevant strategy for advancing the Sustainable Development Goal targets on child health, gender equality, and the elimination of hunger.

## Data Availability

The original contributions presented in the study are included in the article/supplementary material, further inquiries can be directed to the corresponding author.
